# Production of Early Diploid Males by European Colonies of the Invasive Hornet *Vespa velutina nigrithorax*

**DOI:** 10.1371/journal.pone.0136680

**Published:** 2015-09-28

**Authors:** Eric Darrouzet, Jérémy Gévar, Quentin Guignard, Serge Aron

**Affiliations:** 1 IRBI, UMR CNRS 7261, University of Tours, Faculty of Sciences, Parc de Grandmont, 37200 Tours, France; 2 Evolution Biologique & Ecologie, Université Libre de Bruxelles, 1050 Brussels, Belgium; Universidade de São Paulo, Faculdade de Filosofia Ciências e Letras de Ribeirão Preto, BRAZIL

## Abstract

The invasive yellow-legged hornet *Vespa velutina nigrithorax* was accidentally introduced in Europe in the early 2000s. As is the case in colonies of other wasp and hornet species, *V*. *velutina* colonies are known to produce sexuals (males and new queens) at the end of the summer. We show that early-stage colonies in French populations frequently produce males well before the usual reproductive period. The vast majority of the males produced are diploid, which is consistent with the loss of genetic diversity previously reported in introduced populations in France. Since males do not participate in colony activities, the production of early diploid males at the expense of workers is expected to hamper colony growth and, ultimately, decrease the expansion of the species in its invasive range in Europe.

## Introduction

In haplodiploid hymenopteran species, sex is typically determined by one polyallelic locus (single-locus complementary sex determination, or *sl*-CSD) [1–4]. Individuals heterozygous at the sex-determining locus develop into diploid females, while hemizygotes develop into haploid males. However, diploid homozygotes at the sex locus develop into diploid males. Diploid male production (DMP) results in direct fitness costs to parents [4–7]. In a number of species, diploid males experience sterility or reduced survival to adulthood [8]. When fertile and viable, they produce diploid sperm and can father sterile triploid female progeny (but see [9, 10] for examples of diploid males siring diploid female offspring). In social hymenopterans, DMP represents an additional cost because males are produced at the expense of female workers but do not contribute to colony productivity [11, 12]. This phenomenon has been shown to reduce colony growth in bumble bees [13] and to increase mortality during colony founding in ants [11]. Recent theoretical studies have also suggested that DMP can increase the risk of population extinction [14].

Diploid males are rare in large outbred populations because negative frequency-dependent selection maintains a large number of alleles at the sex locus [15]. However, inbreeding, limited gene flow, and genetic drift reduce sex allele diversity and are expected to increase the frequency of diploid males in hymenopteran populations with *sl*-CSD. In particular, invasive species are predicted to suffer from such reduced allelic diversity due to genetic bottlenecks that occur during founding events [16–18]. In line with this, reduced genetic diversity, the loss of sex alleles, and DMP have been observed in introduced populations of the fire ant *Solenopsis invicta* [19] and the European bumblebee *Bombus terrestris* [20].

Native to China, the invasive yellow-legged hornet, *Vespa velutina nigrithorax*, was accidentally introduced to southwestern France around 2004, most likely *via* imported ceramic pottery [21]. The species successfully expanded its range, which now spans over more than 70% of France, and is currently colonizing neighboring countries (Spain, Portugal, Belgium, and Italy) [22–25]. The negative impact of *V*. *velutina* invasion in France is twofold. First, the species preys on several insect and arthropod taxa, thus potentially affecting biodiversity. For example, in southwestern France, *V*. *velutina* is a predator of the domestic honeybee, *Apis mellifera*, and could be induced in colony losses [22–24]. Second, the species presents a risk to human health. Accidents have occurred, some fatal, when people have inadvertently approached the hornet’s nests [26]. Typically, new colonies of *V*. *velutina* are established in the spring by mated queens, after the overwintering period. Colonies first pass through an ergonomic stage by rearing an increasingly large number of workers to ensure colony growth and then produce sexuals at the end of the summer [24, 27]. Males reach the adult stage before the new queens (gynes) (protandry); males and new queens (gynes) emerge in late August/early September and early/mid September, respectively. They take part in reproductive flights; the females then disperse, and the males die.

Recent genetic analyses based on the combination of mitochondrial and nuclear markers support a single introduction event of the yellow-legged hornet in France with a strong founder effect [28]. All populations sampled experience a dramatic loss of genetic diversity. Moreover, the production of “early males” (*i*.*e*., males produced before the end of August) has been observed in a few *V*. *velutina* colonies in France [27]. Early male production has been documented in several social Hymenoptera, but its exact function remains unclear [4, 5, 29–30]. Mating between early males and workers has been seen in orphaned colonies of *Polistes* wasps and allows workers to lay both fertilized and unfertilized eggs [31, 32]. However, such behavior appears to be rare [33, 34] and is viewed as an alternative reproductive strategy that is adopted only when a colony has lost its queen [29, 35, 36]. Another explanation for the production of early males is that they develop from fertilized eggs that are homozygous at the sex locus.

In this study, we examined the production patterns and ploidy of males generated by the invasive hornet *V*. *velutina* in France. We collected colonies during the wasp’s active season to analyze colony composition. We found that significant numbers of males are produced in the spring and early summer, *i*.*e*. before the normal reproductive period. Flow cytometric analyses show that the vast majority of these “early males” are diploid. While the production of early males at the expense of workers during the ergonomic stage is expected to hinder colony growth and, ultimately, the expansion of *V*. *velutina* in introduced populations, the species has spread throughout Europe during the last decade [23, 28]. We propose possible explanations to account for the success of this species in its invasive range.

## Materials and Methods

### Sample collection

Thirty-one colonies of *Vespa velutina* were collected between April and December from 2012 to 2014 in France, mainly in the Indre-et-Loire region (see Table 1 for geographic coordinates). They were brought to the laboratory and kept at -20°C for 48h to kill the hornets. Numbers of queens, workers, gynes, and males in each colony were counted (Table 1).

**Table 1:** Caste composition of 31 *Vespa velutina* colonies collected in France between April and December from 2012 to 2014. Colonies typically produce only workers until the end of summer (end of August) and then produce males and new queens [24, 27]. In the populations sampled in this study, males were reared before the reproductive period. The numbers of workers, gynes (new reproductive queens), and males found in each colony are provided, as are the percentages of males (of all adults) and of diploid males (of all randomly sampled males). Q^+^: queenright colonies; Q^-^: queenless colonies.

Geographic coordinates	Month	Colony/Queen	Males	Workers	Gynes	% Males	Ploidy level of males		% Diploid males
							n	2n	
47°24'09.0"N—0°39'50.0"E	April	C1 / Q^+^	3	3	0	42.86	0	3	100
44°48'29.9"N—0°32'47.0"W	May	C2 / Q^+^	2	0	0	66.66	1	1	50
44°48'29.9"N—0°32'47.0"W	May	C3 / Q^+^	5	5	0	45.45	0	4	100
43°45′25.0″N—0°41′06.0″W	June	C4 / Q^+^	0	1	0	0	-	-	-
43°45′25.0″N—0°41′06.0″W	June	C5 / Q^+^	2	3	0	33.33	-	-	-
43°45′25.0″N—0°41′06.0″W	June	C6 / Q^+^	3	3	0	42.86	-	-	-
47°08′57.0″N—0°10′58.0″E	June	C7 / Q^+^	1	0	0	50	-	-	-
47°15'02.9"N—0°52'40.0"E	July	C8 / Q^-^	28	178	0	13.59	0	12	100
47°24'24.8"N—0°59'09.0"E	July	C9 / Q^-^	0	61	0	0	-	-	-
47°20'17.2"N—0°42'50.0"E	July	C10 / Q^+^	7	19	0	25.93	0	7	100
47°26'17.0"N—0°38'20.0"E	July	C11 / Q^+^	5	71	0	6.49	0	5	100
47°16′41.0″N—0°37′31.0″E	July	C12 / Q^-^	0	72	0	0	-	-	-
47°19'14.2"N—0°55'02.0"E	July	C13 / Q^+^	0	61	0	0	-	-	-
47°15'42.1"N—0°27'58.0"E	July	C14 / Q^+^	8	9	0	44.44	0	8	100
47°33'47.2"N—1°12'53.0"E	August	C15 / Q^-^	1	30	0	3.23	-	-	-
47°15'54.0"N—0°21'09.0"E	August	C16 / Q^-^	21	57	0	26.92	0	12	100
47°21'22.0"N—0°54'34.0"E	August	C17 / Q^+^	0	98	0	0	-	-	-
47°25'18.1"N—0°50'52.0"E	August	C18 / Q^-^	0	244	10	0	-	-	-
47°24'11.2"N—0°36'07.0"E	August	C19 / Q^-^	0	62	0	0	-	-	-
47°35'19.0"N—1°19'39.0"E	August	C20 / Q^-^	6	8	0	38.46	1	5	83.33
47°21'58.0"N—0°43'45.0"E	August	C21 / Q^+^	28	186	1	12.96	0	14	100
47°26'17.0"N—0°38'20.0"E	August	C22 / Q^-^	14	9	0	60.87	0	13	100
47°24'09.0"N—0°39'50.0"E	September	C23 / Q^+^	1	195	0	0.51	1	0	0
47°24'24.8"N—0°59'09.0"E	September	C24 / Q^-^	0	13	0	0	-	-	-
47°23'34.1"N—0°41'01.0"E	September	C25 / Q^+^	63	86	2	41.45	0	23	100
47°23'34.1"N—0°41'01.0"E	September	C26 / Q^+^	57	80	0	41.3	0	24	100
47°02'31.9"N—0°49'08.0"E	October	C27 / Q^+^	106	150	0	41.25	-	-	-
47°19'14.2"N—0°55'02.0"E	November	C28 / Q^-^	162	96	50	52.6	5	5	50
47°54'14.0"N—1°54'26.0"E	November	C29 / Q^-^	3	2	2	42.86	1	2	66.67
47°19'58.1"N—1°02'57.0"E	December	C30 / Q^-^	10	8	10	35.71	10	0	0
47°14'12.8"N—0°07'40.0"E	December	C31 / Q^-^	33	44	108	17.83	4	0	0

Our field study did not involve endangered or protected species. Therefore, no specific permissions were required to collect hornets in France. Rather, as *V*. *velutina* is an invasive species, the French government recommends the elimination of colonies (DGAL/SDSPA/N2013-8082, 10 May 2013).

### Ploidy analysis

**Fig 1:**
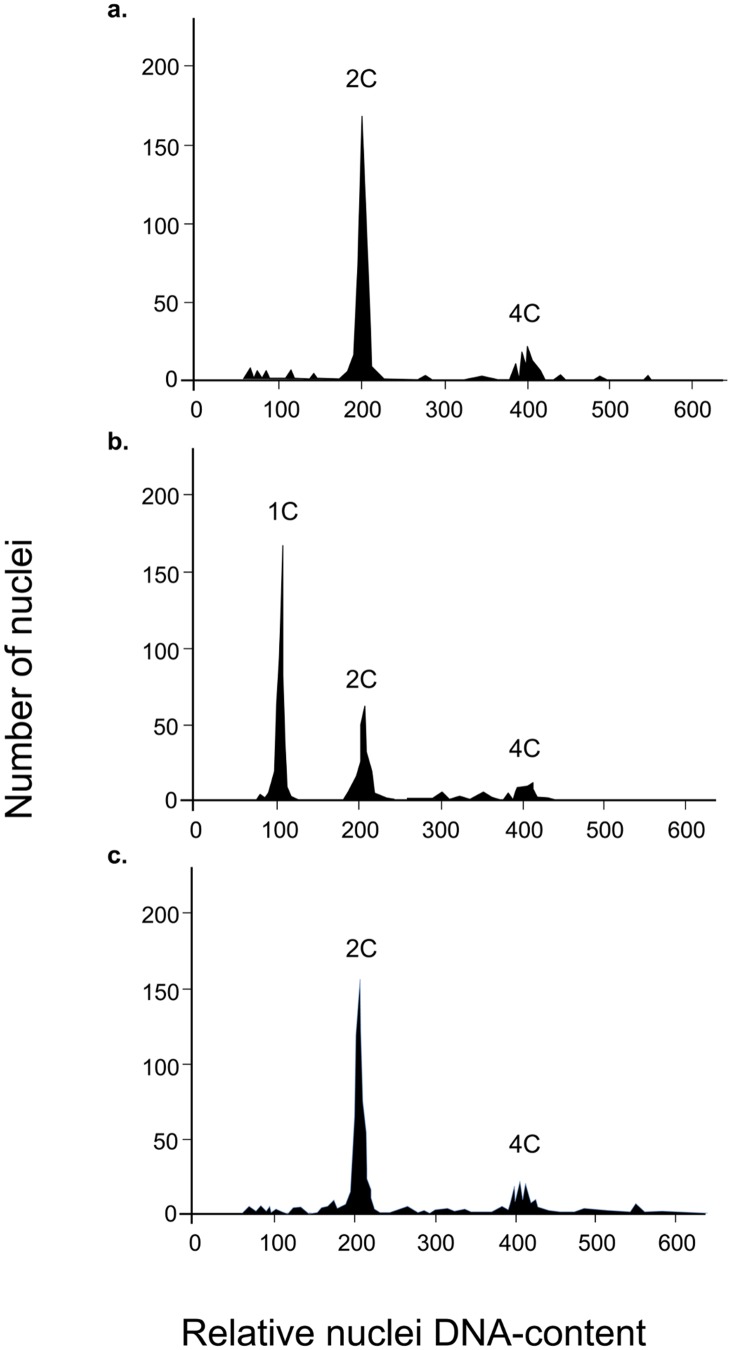
Flow cytometric DNA histogram of diploid female (a), haploid male (b) and diploid male (c). Each histogram shows the nuclear frequency with regard to DNA content for the head of a single individual. The first peak corresponds to ploidy level, the second peak to nuclei with a double DNA content and the third peak to polyploid nuclei. In haploid males, the second peak (2C) corresponds to nuclei from mandibular muscles where cells are diploids.

The ploidy level of the *V*. *velutina* males sampled was determined by flow cytometry [5, 37]. Colonies and individual males were randomly sampled. Samples were prepared by pulverizing the head of each individual hornet in a 1-ml 4′,6-diamino-2-phenylindole dihydrochloride (DAPI) solution (CyStain) with a pestle. The suspension was subsequently filtered using CellTrics (mesh size: 20 μm). Flow cytometric analyses were performed using a PA-I flow cytometer (PARTEC, Partec Gmbh, Münster, Germany) equipped with a UV-LED 365 nm light source and employing an optical configuration described elsewhere [38]. For each sample, the ploidy of 2,500 nuclei was analyzed. A threshold on FL2-A was used to exclude very small debris. We used flow cytometric DNA histograms of known haploid males and diploid females as references to determine the ploidy of unknown males (Fig 1).

## Results

Several *V*. *velutina* colonies produced males throughout the entire season, not just at the end of the summer (Table 1). Fifteen of the 22 colonies (68%) collected from April to August (*i*.*e*., before the reproductive period) contained early males. These males accounted for between 3 and 67% of colony members. Flow cytometric analyses revealed that more than 97% (84/86) of the early males were diploid; only two males were haploid.

Of the 9 colonies collected during the reproductive period (September–December), 8 contained mature males. These males represented between 0.5 and 53% of colony members. Of the 75 males sampled, 54 (72%) were diploid. The remaining 21 were produced by arrhenotokous parthenogenesis and were haploid. Two colonies (C30 and C31; Table 1) produced only haploid males.

The very high proportion (>50%) of diploid males produced among offspring in colonies C2, C22 and C28 likely results from sampling bias, e.g. due to workers being not collected because they were foraging or some died before sampling.

## Discussion

This study shows that colonies of the invasive yellow-legged hornet *Vespa velutina nigrithorax* in France (1) produce males throughout the species’ active season, even well before the reproductive period, and (2) that most of these males are diploid. The predominance of diploid males is consistent with the genetic bottleneck experienced by this species following its introduction into France [28]. Similarly high levels of diploid males have been observed in the fire ant, *Solenopsis invicta*, in its introduced range in North America [11]. DMP is attributable to a loss of allelic diversity at the sex-determining locus [39]. The number of sex alleles in *V*. *velutina* remains unknown. Estimates based on the frequency of diploid males in populations suggest that the effective number of alleles at the sex-determining locus varies greatly within Hymenoptera, with an average of 5 alleles in *Halictus poeyi* [40], 15 in *Solenopsis invicta* [39], 19 in *Apis mellifera* [41], 20 in *Melipona compressipes fasciculate* [42], 24 or more in *Bombus terrestris* [43], and 33 in *Polistes chinensis antennalis* [30].

In social Hymenoptera, DMP may severely hamper colony growth because a large percentage of diploid eggs yield males instead of females [11, 14]. First, a colony may lose out if queens produce diploid males instead of workers during the ergonomic stage because males do not benefit the colony, in contrast to workers (*e*.*g*., building the nest and feeding larvae). Of the offspring produced by queens who mate with a single male carrying the same sex allele, 50% will be diploid males. Field studies in France have found that *V*. *velutina* nests may be abandoned early on, which suggests that colony founding has failed [44]. Second, diploid males impose particularly high fitness costs on the colony since they are usually sterile. Even when they are not, they produce diploid sperm and father sterile, triploid female progeny (reviewed in [8]). The production of triploid offspring, both females and males, has indeed been observed in various hymenopteran species, including *Athalia rosae* [45], the parasitoid wasp *Cotesia vestalis* [46], the bumblebee *Bombus terrestris* [47] or the ant *Tapinoma erraticum* [5]. A triploid male was also found in *V*. *velutina* (unpublished data). These findings suggest that diploid males produce diploid sperm and father triploid offspring.

In introduced species, founder effects and genetic drift can reduce the genetic diversity as populations are becoming established. They may result in inbreeding depression, a main contributor to population extinction [48–50]. In haplodiploids, the production of nonviable or sterile diploid males due to inbreeding is expected to reduce population growth rates and effective sizes, potentially creating a rapid extinction vortex [18, 51]. Remarkably, while DMP is predicted to affect the expansion range of introduced *V*. *velutina* populations, the species has spread throughout Europe during the last decade [24,28]. This indicates that the yellow-legged hornet can establish successful populations, even from a limited number of foundresses with low genetic variability [28].

Several biological and environmental factors may have contributed to the success of this invasive species in Europe, including favorable climatic conditions [52], the abundance of preys (honeybees), multiple mating by queens [28] and occasional production of early haploid males. If DMP has a large negative impact on colony foundation and survival, there may be a selective advantage for queens to mate with multiple partners to reduce the likelihood of producing diploid males [53, 54]. Recently, genetic analyses of French populations of *V*. *velutina* have revealed that queens mate with an average of 4.6 males (SD = 2.3; [28]). Interestingly, this value is higher than that observed in other *Vespa* species [28, 55]. To date, queen mating frequency and diploid male production in native populations of *V*. *velutina nigrithorax* remain unknown. Whether multiple mating has been selected for in invasive populations to reduce the costs associated with DMP and/or the probability of mating with diploid males remains to be studied. Our data also show that *V*. *velutina* colonies rear a small number of haploid males before the reproductive season. The function of these early males is enigmatic. One hypothesis is that early males could mate with virgin reproductive females (gynes) that survive the winter. Another, non-exclusive hypothesis is that early males mate with workers from orphaned colonies; mated workers could then become new queens and leave their natal nests to found new colonies. Both these scenarios illustrate alternative reproductive strategies adopted by gynes and workers [29, 33, 56–59] and may have contributed to the rapid expansion of *V*. *velutina* in Europe.

Clearly, future research should explore (1) the reproductive function of early males and their potential role in alternative female reproductive strategies, (2) the inbreeding coefficient resulting from nonrandom mating within invasive populations, (3) whether diploid males father triploid fertile females and, more generally, (4) the ecological consequences of DMP for populations of the invasive hornet *V*. *velutina* in Europe.
